# The power within: Mass media, scientific entertainment, and the introduction of psychical research into China, 1900–1920

**DOI:** 10.1002/jhbs.22236

**Published:** 2022-12-08

**Authors:** Luis Fernando Bernardi Junqueira

**Affiliations:** ^1^ History Department University College London London UK

**Keywords:** Chinese psychology, hypnotism, modern China, popular science, psychical research, spiritual science, spiritualism, stage hypnotism

## Abstract

How did a new science initially promoted by only a few individuals eventually become a widespread cultural phenomenon practiced and known by thousands of people? Following a transnational approach, this article traces the introduction of psychical research into China during the first two decades of the 20th century. Known in the Republican period (1912–1949) as Spiritual Science (*xinling kexue* or *xinling yanjiu*), psychical research flourished between the 1920s and 1930s, playing a key role in the popularization of applied psychology and mind‐cure across China. This article takes a step back from the heyday of Spiritual Science by looking at the period that immediately preceded and helped define it. Focused on wide‐circulation newspapers, popular manuals, and stage performances, it teases out the ways in which Chinese popular culture translated European, American, and Japanese psychical research to local Chinese audiences in the midst of China's search for modernity. By naturalizing the reality of psychic powers, spiritual scientists blurred the boundaries between science and superstition in a period when these were posited as diametrically opposed.

## 1 INTRODUCTION

This article examines the introduction of psychical research into China during the first two decades of the 20th century. Emerging in late‐19th‐century Europe, psychical research was a transnational field of study concerned with the investigation of abnormal phenomena such as hypnotism, clairvoyance, mediumship, mind‐reading, and faith‐healing. Over the past few years, historians have disputed previous teleological claims about the triumph of science over occult and spiritual matters by showing how the origins of modern academic disciplines—such as psychology and anthropology—were inseparable from the activities of those involved in psychic studies (Lachapelle, 2005; Mülberger, 2016; Sera‐Shriar, 2022; Shamdasani, 2015; Sommer, 2012, 2013a; Treitel, 2004). The fascination such phenomena exerted over “physical‐psychical scientists” in fin‐de‐siècle Britain, for example, helped shape the boundaries of modern physics and psychology in a time when the threshold between “normal” and “supernormal” was being constantly contested (Noakes, 2019; Raia, 2007; Sommer, 2013b). And beyond the scientific laboratory, a plethora of popular books, newspapers, and performances were readily available to anyone interested in paranormal adventures (Gauld, 1992; Ichiyanagi, 2021; Lachapelle, 2015; Pablo, 2003; Priori, 2014).

Indeed, it may come as a surprise for many to learn that psychical research also blossomed in early‐20th‐century China. Not only did it play a key role in the popularization of applied psychology and mind‐cure across the country, but also paved the way for the *qigong* boom between the 1970s and 1990s (Palmer, 2007) and the Body‐Mind‐Spirit (*shen‐xin‐ling*) movement today. Strikingly, despite a copious volume of primary sources, Chinese histories of psychology have barely acknowledged the “hypnotism craze” that swept across Republican China (1912–1949). Preliminary research suggests that from the early 1900s to the late 1940s, well over 300 specialized books, 5000 articles, and 20,000 advertisements for hypnotism and mind‐cure were published in China alone, many of which were translated from, or inspired by works originally printed in Japan, Europe, and the United States. This number is far more expressive than anything Chinese academic psychologists produced over the same period, shedding light on the wider—yet hitherto unexplored—Chinese appropriations of psychic studies. Historians have only recently begun to scratch the surface of Chinese psychical research (Zhang, 2020; Zheng, 2018); much has yet to be investigated, such as the movement's transnational dimension, its reappraisal of science and superstition, as well as its use of the psy‐disciplines to build an ideal “modern China.”

Called “Spiritual Science” (*xinling kexue*/*xinling yanjiu*) in Chinese, psychical research grew out of spiritualism, hypnotism, and the wave of mind‐cure movements that took over the world between the late 19th and early 20th centuries (Harrington, 2008; Hickey, 2019; Yoshinaga, 2007). Much of its appeal on Chinese soil stemmed from the ripening of psychical research in early‐20th‐century Japan. During the Meiji era (1868–1912), Japan set out on a course of rapid change following Western models, which introduced into the country the latest scientific discoveries from Europe and the United States—including psychical research, hypnotism, and the Ouija board (Ichiyanagi, 1997, 2021; Wu, 2020; Yoshinaga, 2006). By contrast, China, which had for centuries been respected as the political and cultural center of East Asia, was on the verge of fragmentation and social collapse.

The early 20th century marked a dramatic turning point in Chinese history. Humiliated by foreign imperialism and ravaged by civil war, the last Chinese dynasty fell in 1911, putting an end to over 2000 years of imperial rule and throwing the country into decades of social turmoil. Chinese reformers agreed that to survive, China had to be modernized; and to become modern, superstition should be eradicated. The urge to reconstruct the Chinese mind culminated in the May Fourth and New Culture movements of the 1910s and 1920s, which aimed to replace traditional Confucian values with a new overarching authority: science. Hailed as the epitome of modernity, science ultimately became the standard against which all other forms of knowledge ought to be judged (Shen, 2016). This “paradigm shift” (Lagerwey, 2019) led to a series of antisuperstition campaigns across the country (Nedostup, 2009), with heated debates taking place in the Chinese press regarding what, in practice, constituted superstition and the most effective methods to eliminate it (Huang, 2015). It was within this chaotic period that psychical research was introduced into China, thriving through the efforts of Chinese elites committed to science and social reform yet dissatisfied with purely materialistic views of reality.

To Chinese intellectuals, the Meiji reforms served as an example to be emulated, and by the early 1900s, waves of Chinese students were being sent to Japan. While studying such modern subjects as law, medicine, and social sciences in Japanese universities, a few young Chinese came across the discipline of psychical research. Their enthusiasm for this “revolutionary science” rapidly materialized into hundreds of articles and advertisements published in the Chinese popular press—with their demonstrations of the “remarkable effects” of hypnotic phenomena attracting vast crowds of spectators. Drawing on foreign sources and scientific authorities, those students explained how psychical research complicated the boundaries between the natural and the supernatural, between mind and body, offering a fresh perspective on the future of science and China's path to modernity.

The Chinese interest in psychical research was directly linked with the growth of modern professions in early‐20th‐century China—particularly in the fields of publishing, healthcare, and education—and it remained largely an affair of the urban middle classes. Established in the mid‐to‐late 1910s, organizations such as the Chinese Institute of Mentalism (CIM; *Zhongguo xinling yanjiuhui*), Chinese Hypnotism Association (CHA; *Zhongguo jingshen yanjiuhui*), and the Society of Spiritualism (SoS; *Lingxuehui*) were for most of their time headquartered in Shanghai's foreign concessions.^1^ Contrary to the short‐lived SoS, whose few supporters tended to be older, the influential CIM and CHA grew to thousands of members between 20 and 30 years old, most of whom were teachers, writers, publishers, military officers, psychologists, and medical doctors. The CIM and CHA would continue to operate until the early 1940s, enjoying their heyday in the 1920s and 1930s through a profusion of publications, performances, and healthcare services around hypnotism and mind‐cure.

Taking a step back from the zenith of Spiritual Science, this article will examine the period that immediately preceded and helped define it. In contrast to the publications and activities by CIM and CHA, which targeted a specialized audience, the sources analyzed below will illuminate the appropriation of psychical research through popular media directed at those still unfamiliar with the subject. Moreover, unlike the vocal community of spiritual scientists that would emerge in the 1920s, most of the writers herein discussed did not identify themselves as psychical researchers but rather as supporters and sympathizers of the field. How did a new science initially promoted by a few individuals eventually become a widespread cultural phenomenon practiced and known by thousands of people? Focused on the introductory phase of psychical research in China (c. 1900–1920), this article will clarify its popularization through (1) wide‐circulation newspapers, (2) popular books, and (3) stage performances. Pursuing a transnational approach, it will indicate how the introductory phase laid the foundation for the consolidation of Spiritual Science in the 1920s. More broadly, it aims to demonstrate the contribution of Spiritual Science and its diffusion process to the transnational histories of psychology, knowledge‐making, and popular science.

## 2 PSYCHICAL RESEARCH IN CHINESE NEWSPAPERS

### 2.1 The Afterlife

In his groundbreaking book, *Physics and Psychics: The Occult and the Sciences in Modern Britain*, Richard Noakes (2019) observed how the popular press became a vital channel through which fin‐de‐siècle British scientists and intellectuals introduced the principles and social uses of psychical research to broader audiences. Newspapers, magazines, and popular science books enabled psychical researchers to express their views about controversial subjects—such as the relationship between religion and science, the causes of psychic phenomena, and the reality of the soul—with a degree of freedom not allowed in scientific journals. Hirotaka Ichiyanagi's study of Meiji Japan has also indicated the key role the popular press played in the diffusion of hypnotism, spiritualism, and psychical research across the country (1997, 2021). It is, therefore, not surprising that newspapers also became an important doorway through which psychical research was introduced to Chinese readers.

Although articles on psychical subjects could be found across all genres of Chinese periodicals—from women's and education magazines to medical and political journals—no media vehicle engaged so ardently in the introduction of psychical research than the *Eastern Miscellany* (*Dongfang zazhi*). Printed by the Commercial Press from 1903 to 1948, *Eastern Miscellany* stood among the most influential magazines of 20th‐century China (Tao, 2013). It assumed a central role in the introduction of scientific ideas into the country, and during the editorial command of Du Yaquan (1873–1933) between 1911 and 1920, the magazine attended closely to issues of selfhood and psychology, publishing well over 50 articles on such subjects as hypnotism, mediumship, telepathy, clairvoyance, faith healing, dreams, experimental psychology, the survival of the soul, scientific Buddhism, and the relationship between science and religion. Most of these consisted of loose translations from English, German, French, and Japanese into vernacular Chinese. Not only did those articles help the Chinese public familiarize themselves with the principles of psychical research but also crafted a vocabulary readily available to spiritual scientists in the 1920s.

Multiple scientific investigations conducted by members of the Society for Psychical Research (SPR) and the American Society for Psychical Research (ASPR) dominated the magazine's pages devoted to psychic studies. This made names like Oliver Lodge (1851–1940), William Crookes (1832–1919), William F. Barrett (1844–1925), William James (1842–1910), and Frederic W. H. Myers (1843–1901) gradually familiar to Chinese readers. Likewise, eulogizing expressions such as “latest science” (*xin kexue*), “latest explanations” (*xin jieshi*), and “latest discoveries” (*xin chuxian*) presented psychical research as a scientific novelty that, if properly mastered by the Chinese, could contribute to the modernization of China just as it had done in America, Europe, and Japan. The field, it was claimed, had the potential to expand the horizons of human knowledge in an unprecedented manner, and the Chinese should not fall behind in this enterprise.

The advanced technological arsenal that permitted the Great War (1914–1918) carnage had a profound impact on the minds of American, European, and East Asian intellectuals, many of whom had previously venerated science as a source of progress and the savior of mankind. In China, discontent with the course of Western modernity began to attract an expanding community of sympathizers by the 1910s. This became particularly evident among political revolutionaries disenchanted with the collapse of the newly established Republic and the destruction witnessed in Europe during WWI. Scientific materialism had failed, or as Peter Zarrow puts it, the “Chinese judged the West by its own standards and condemned it” (Zarrow, 2005, p. 158). The revival of spiritualism in Europe and the United States during the interwar years is often described as a reaction to the potentially destructive nature of scientific rationalism and its contempt for religion and morality (Hazelgrove, 1999; Lamont, 2012). Likewise, the growing appeal that redemptive societies and transnational networks exerted over Republican Chinese elites revealed not a rejection but a new engagement with science—an engagement also inspired by the activities of Western and Japanese psychical researchers.

*Raymond, or Life and Death* (1916) was a typical product of this period. Authored by the renowned British physicist Oliver Lodge, the controversial book caused a sensation in the British and international press (Ferguson, 2020). It recounts how Lodge's son, Raymond, who died during the Great War, returned to the family through the mouth of spiritualist mediums. The memoir reveals Lodge's highly positive account of postmortem existence, and his international fame as an accomplished physicist soon turned *Raymond* into a global best‐seller. Excerpts of *Raymond* began to come out in the Chinese press only a few months after its original edition was published in 1916. In a series of articles on spiritualism and psychical research published in 1917, the young political activist and future member of the Chinese Communist Party, Hu Yuzhi (1896–1986), introduced to Chinese readers two pieces originally authored by the British psychical researcher John A. Hill (1872–1951). The first article comprised a translation of the section “Investigation: Methods and Examples” of Hill's book *Psychical Investigations* (1917), wherein the author proposes “personal experience” as an effective method to investigate spiritualist claims (Hu, 1917a). The second article focused on Lodge's *Raymond*. Its Chinese translation offers an overview of Lodge's life, the book's content, the controversies it sparked within British society, and its value for psychical research. It hails Lodge as one of the greatest physicists of his time, describing the several spiritualist séances he conducted with the assistance of mediums. After years of investigation in psychical research, the article explains, Lodge became a firm believer in the afterlife, and his achievements in electromagnetic waves and wireless telegraphy also inspired him to theorize about the existence of an “invisible world beyond our material one” (Hu, 1917b).

Lodge's commitment to reconciling science and religion was restated in another Chinese article: “A New Explanation for the Afterlife” (*Weilai shenghuo zhi xinjieshi*). It consists of an introduction to *Claude's Book* (1918), a collection of mediumistic conversations with the soul of Claude H. Kelway (1895–1915), another young British soldier killed during WWI, through the mouth of the medium Mrs. Osborne Leonard—the same who had helped Lodge contact his deceased son a few years earlier. Edited by Claude's mother, *Claude's Book* received a highly positive review from Lodge, which was printed on the first page of its English edition and, as the Chinese translator disclosed, was decisive to attract his attention. “Thanks to eminent intellectuals like Arthur C. Doyle and Oliver Lodge, alongside the millions of copies that their books have sold worldwide, [Britain has become] the unrivaled center for psychical research (*guixue*; lit. ‘ghost studies’) in the world.” Lodge's scientific endeavor to “use the language of the spirits to clarify the physical reality of the otherworld,” the anonymous Chinese translator added, “suffices to encourage us to venture into these otherwise neglected subjects” (“Weilai shenghuo,” 1920).

The *Eastern Miscellany* extolled Lodge's “outstanding empirical studies on mediumship (*lingjiao*) and telepathy (*gantong*)” in another translated article originally authored by the American theologian and Christian reformer Lyman J. Abbott (1835–1922). Drawing the fashionable analogy between wireless telegraphy and telepathy, Abbott asked: “How is it possible to transfer formless thoughts from one mind to another? […] Just as nobody today would claim that a metallic line is necessary for a telegraph to be transmitted, why should we assume that the communication between two minds (*xinling*) must be based on a material conductor?” Lodge's latest achievements in physics and psychical research (*xinli kaoyan*), Abbott concluded, have proved that “reductive materialism has no longer a place in modern scientific thought.” It was time to emulate Lodge and investigate the biggest question of humanity: “Is there life after death?” (Abbott, 1918). The insights electromagnetism and wireless telegraphy yielded for the scientific study of telepathy and clairvoyance would become central for spiritual scientists, proving that psychical research was not a denial but rather an advanced form of science no longer constricted to purely material pursuits.

The danger of “materialistic science” (*wuzhi kexue*)—the assumption that everything in the universe is mere matter and motion—was a central theme in Chinese translated articles on psychical research. “The revival of spiritualism (*linghun zhuyi*) and psychical research (*guixue*) across Europe,” a Chinese writer asserted in 1920, “is a consequence of the Great War butchery, [which] destroyed millions of lives and revealed the fallacies of material progress and materialistic science” (“Weilai shenghuo,” 1920). And to the materialistic minds who despised telepathy as deception, Abbott ironically asked: “In the history of experimental science, we know that in the beginning scientists ridiculed vehicles and steamships as naïve dreams, the telephone as a child's toy and the electric lamp as mere ornaments. They even denounced wireless telegraphy as wild fancy. They assured all these inventions would eventually fail. But after all these years, could we say that their claims have succeeded?” (Abbott, 1918). In the decades that followed, Abbott's argument would be frequently invoked by spiritual scientists as a counterattack to those who criticized psychic endeavors as superstition or pseudoscientific.

The widespread claims for the advancement of Western science to the detriment of religion and traditional thought during the New Culture and May Fourth movements led to a surge of articles exposing the “materialist malaise” that was taking over Chinese society (Zarrow, 2005). In a series of articles for the *Eastern Miscellany*, the philosopher, political activist, and most vocal exponent of Nietzsche's thought in Republican China, Li Shicen (1892–1934), criticized what he called the “bankruptcy of science” (*kexue pochan*), which had failed to improve morality and provide answers for the biggest questions of life. But the limitations of materialistic science, Li continued, had encouraged “a wave of modern scientists to move from a negative to an increasingly more positive attitude towards religion [as expressed] in the inception of psychical research (*jingshen tanjiu*) […] Phenomena scientific orthodoxy had been unable to explain, like clairvoyance, automatism, thaumaturgy, ghost‐hunting, telepathy, spiritualism, and the subconscious have all been recently taken up by psychical researchers” (Li, 1921).

Starting with William James's theory of pragmatism and philosophy of religion, Li outlined the achievements of other prominent psychical researchers such as William Crookes, Nicolas Camille Flammarion, Cesare Lombroso, and Oliver Lodge. Supporter of reformed Buddhism, Li believed psychical research could demonstrate that science and religion were not opposed to each other—and that Buddhism had much to contribute to their reconciliation (Li, 1923). Following the views of Japanese Buddhist reformers such as Inoue Enryō—to whom we will return later—Li held that Buddhism's analytical approach and its conception of mind were largely aligned with the latest scientific developments in psychology and psychical research (Li, 1920). Indeed, by the 1920s spiritual scientists were drawing heavily on Buddhist vocabulary and meditation methods as a means to explain the experience and efficacy of hypnotism—an approach directly imported from Japan (Ichiyanagi, 1997, pp. 72–75; Wu, 2019). Erik J. Hammerstrom's (2015) study of how early‐20th‐century Chinese Buddhists reexamined their tradition through the lens of science, particularly psychology, suggests that Li belonged to a much larger community of Chinese reformers discontent with the materialistic values Western modernity embodied.

Chinese newspapers portrayed psychical research as a new way of conceptualizing and making science. Echoing the voices of Western psychical researchers, Chinese writers hailed the potential the physical sciences—like physics, chemistry, and biology—had for the investigation of psychical phenomena (ex., “Shijie shenmi”, 1916). These views are neatly summarized in another article Hu Yuzhi wrote for the *Eastern Miscellany* in 1921, “Superstition and Modern Thought” (*Mixin yu jindai sixiang*). The piece is a selected translation of the bestselling *Modern Science and Modern Thought* (1885), authored by the British scientific writer Samuel Laing (1812–1897). The book is a criticism of spiritualist mediums, whom Laing denounced as “professional conjurers who manufacture ghosts” (p. 232). On the surface, Laing's view seems to resonate with that of Chinese intellectuals who blamed the Chinese belief in the supernatural on the country's lack of scientific education (Huang, 2015). But Laing's argument is not as simplistic as it seems, and that might be the reason Hu picked that particular section of the book. While Laing did denounce the “fallacy of Spiritualist communications,” Hu explains, “he also praises those men of a very different order [like William Crookes and Alfred R. Wallace] who understand what scientific evidence really is [and thus] allow phenomena such as mesmerism and clairvoyance to turn into valuable fields for scientific investigation.” Simply put, Laing held that the phenomena whose cause “naïve Spiritualists” attributed to disembodied spirits could be properly explained in psychological and physiological terms. A close reading of the English text and its Chinese translation suggests that Hu intended to use this article as a criticism of the SoS, which by 1921 was already renowned for its advocacy regarding the existence of spirits (Zheng, 2018).

The growing interest in psychoanalysis in Republican China also introduced to Chinese readers neologisms and subjects with a close affinity to psychical research (J. Y. Zhang, 1992). Key concepts like the unconscious and subliminal consciousness featured prominently in the publications of the *Eastern Miscellany*. In a special edition dedicated to the oeuvre of the German biologist and psychical researcher Hans Driesch (1967–1941), the magazine invited him to outline the latest studies of the unconscious in Europe. Together with the philosopher and psychical researcher Henri Bergson (1859–1941), Driesch's vitalist philosophy had an enormous impact on the minds of early Chinese reformers (Chang, 2017). His lengthy article explains a variety of psychical phenomena, including hypnotic anesthesia, suggestion, hallucinations, spirit‐materialization, clairvoyance, telepathy, automatic‐writing, and abnormal psychology. Once ignored by orthodox science, Driesch clarifies, “these subjects have been recently taken up by prominent scientists who developed the discipline of Parapsychologie (*guaiyi xinlixue*) in Germany, also known as Metapsychique (*chaoxinlingxue*) in France, or Psychical Research (*xinling yanjiu*) in Great Britain.” Their scientific investigations have not only “determined the reality of the unconscious but also revealed that our minds are not limited to our physical bodies” (Driesch, 1923).

Most articles about psychical research published in the *Eastern Miscellany* were Chinese translations of popular publications authored by members of the SPR and ASPR. Likewise, when Spiritual Science organizations began to emerge in Shanghai in the late 1910s, they promptly posited themselves as the “siblings” of their British and American counterparts. Even critics of spiritualism, like the psychologist and former president of Peking University, Chen Daqi (1886–1983), admired the experiments conducted at those foreign institutions (1919). Indeed, it is the simplified version of an article William Barrett published in the *Contemporary Review* in January 1918 that best illustrates how translations helped define the boundaries of Spiritual Science in China. Published in the *Eastern Miscellany* in September 1918, the Chinese translation summarizes the reasons for the foundation of the SPR in 1882, as well as the methods and objects of study of psychical researchers:

1. The alleged action of mind upon mind, independently of the recognized channels of sense; thought‐transference, now included in the wider term telepathy.
2. The phenomena of hypnotism.
3. The transcendental perceptive power, or clairvoyance, asserted to exist in certain sensitives.
4. First‐hand evidence for veridical (truth‐telling) apparitions of the living or dying.
5. The alleged physical phenomena of spiritualism.
6. Historical evidence bearing on these subjects (tr. Barrett, 1918; Luo, 1918).

Barrett's six areas of concern roughly comprise what Yu Pingke, the eventual director of the CIM, would announce in 1922 as the “six central issues of Spiritual Science.” “The aim of the Society,” Barrett continued, “is to approach these various questions without prejudice, and in the same spirit of exact and unimpassioned enquiry which has enabled science to solve so many problems, once not less obscure nor less hotly debated.” After exemplifying how recent discoveries in physics could benefit psychic studies, the Chinese translation ended by summarizing the five main principles of psychical research according to Barrett:

1. The physical place is not the whole of Nature, nor the outer conscious self the whole of our human personality.
2. Within us are highly capacious powers, now subject to the temporary limitations imposed by our bodies.
3. Our mind can act independently of the material brain, and therefore in all probability can survive.
4. There is a spiritual world [beyond the material one] wherein active life and intelligence exist.
5. [Death is not the end of life,] but the introduction to a larger life and an infinite hope (tr. Barrett, 1918); Luo, 1918).^2^

Alongside the “six concerns,” Yu (1922) would hail Barrett's five principles as the foundation of Spiritual Science.

### 2.2 Telepathy, clairvoyance, and thoughtography

In 1882, Frederic W. H. Myers and his fellows at the SPR coined “telepathy” as a collective term to cover “all cases of impression received at a distance without the operation of the recognized sense organs” (Barrett & Myers, 1882, p. 147). The neologism should replace the older “thought‐reading” and “thought‐transference” largely associated with magicians and popular performers at the time (Luckhurst, 2002, pp. 68–71). Whereas its related term, “clairvoyance,” has its roots in old French, mid‐20th‐century spiritualism gave it a new lease of life. Myers defined it as “the faculty or act of perceiving, as though visually, with some coincidental truth, some distance scene,” but admitted that, in practice, the boundaries between telepathy and clairvoyance remained blurred (Myers, 1903, p. xvi). From its foundation in 1882 to the turn of the century, the core of SPR's activities revolved around scientific investigations of telepathy/clairvoyance (Luckhurst, 2002), whose achievements also inspired psychical researchers in Japan and China.

The hypnotism craze that took over fin‐de‐siècle Japan was immediately followed by reports of Japanese, both men and women, capable of displaying remarkable psychic powers under hypnotic influence. While some asserted being able to communicate with the souls of the dead, others could read with their ears, see through solid matter, transmit information independently of the recognized channels of sense, and even imprint mental images onto photographic plates (Ichiyanagi, 1997, 2021, pp. 80–143). Each of these abilities gained a proper name—usually derived from Classical Chinese—being commonly subsumed under the collective term *senrigan* (Chinese: *qianliyan*), which eventually became the standard translation of the English “telepathy” and “clairvoyance.” The meaning of *senrigan*/*qianliyan*, however, is broader than its English counterparts and deeply rooted in Chinese popular culture.

In Chinese religion, Qianliyan (Eyes‐that‐See‐Thousand‐Miles) is the name of a sea god who together with Shunfeng'er (Ears‐that‐Hear‐with‐the‐Wind) have long acted as guardians of the temples of the sea goddess Mazu. While Qianliyan's sharp vision was believed to protect sailors at night and during adverse weather conditions, Shunfeng'er's acute hearing helped them distinguish favorable winds from potential storms. Originally from southern China, both gods became increasingly known across the country and abroad through novels such as *Journey to the West* (1592) (Ptak, 2017). More broadly, *qianliyan* also indicated humans with exceptional visual capacities or the abilities themselves—as personified in the divine figure of Qianliyan. These multiple interconnected meanings might explain why the term was so extensively invoked in the Japanese and Chinese press to convey abilities like telepathy and clairvoyance. By adopting the name of a well‐known Chinese god whose all‐seeing powers resembled those described in the latest Western scientific writings, Japanese—and later Chinese—enthusiasts sought to familiarize their countrymen with the “newest science” of psychical research while showing that the sort of phenomena it studied were no novelties in their respective countries.

The wide adoption of age‐old terms in the East Asian context contrasts sharply with the Anglo‐American case. As Roger Luckhurst has noted, Myers and his SPR fellows deliberately coined the term telepathy so as to “exclude the spirit hypothesis,” that is, to distance their laboratory studies from any connection to séances or the magician's stage (2002, pp. 70–71). In Japan and China, on the other hand, even though apparently more neutral neologisms like *tōshi* (Chinese: *toushi*; lit. “see‐through”) were favored by academic‐oriented researchers, these terms did not take off as the older—and apparently more religious—*senrigan*/*qianliyan*. While Western psychical researchers tried to distance themselves from the past, Japanese and Chinese spiritual scientists seem to have pursued a more flexible approach toward tradition—or were possibly persuaded to concede in face of the enormous popularity *senrigan*/*qianliyan* was already enjoying among the population at large. Another factor is that while many Western psychical researchers were academics, Spiritual Science was chiefly an affair of lay individuals whose support stemmed from the general public.

The new meanings Japanese scholars ascribed to *senrigan/qianliyan* began to integrate the Chinese vocabulary by the early 1910s, and as studies of telepathy and clairvoyance developed worldwide, so did the category of *qianliyan* in China. A series of articles for the *Eastern Miscellany* published between 1913 and 1915, for example, explains the difference between *qianliyan*, *qiannianyan*, and *toushi*. While the first indicated the ability to gain information about events happening far away in space, the second referred to the same ability yet related to events occurring far away in time. *Toushi*, on the other hand, was the capacity to identify things hidden in front of one's eyes through extrasensory perception (Wang, 1914; **​**Zhang, 1913). The Buddhist and medical reformer Ding Fubao (1915) held a similar view yet employed a more descriptive terminology. After consulting the Japanese translation of an unknown German work, Ding divided *qianliyan* into three categories: *toushi* (Hellsehen, or clairvoyance), *kongjian de yuangeshi* (Raumliehes Fernsehen, or spatial remote‐viewing), and *shijian de yuangeshi* (Zeitliches Fernsehen, or temporal remote‐viewing). But not everyone was satisfied with the usage of *qianliyan*. In 1911, a Chinese reporter declared that *qianliyan*, “also known as the science of the spirit (*shenxue*),” referred to nothing but what Buddhists had long called *tianyantong*, the ability of “all‐seeing” and one of the six *abhijñā* (higher forms of knowledge) that could be achieved through meditation (“Qianliyan,” 1911).

In the early 1900s, reports of individuals who displayed exceptional powers under hypnotism sparked a furor across the Japanese media and soon attracted the attention of the Japanese academic world (Ichiyanagi, 2021; Nagayama, 2005). Around 1910, Fukurai Tomokichi (1869–1952), a professor of psychology at Tokyo Imperial University and one of Japan's earliest psychical researchers, launched a series of investigations into such phenomena. It was during his experiments with the Japanese medium Nagao Ikuko (1871–1911) that Fukurai discovered *nensha* (thoughtography), the ability to imprint mental images onto photographic plates. Although Fukurai's experiments caused much controversy and eventually forced him to resign from his academic position, his lifelong commitment to studies of telepathy, clairvoyance, and thoughtography, as well as his close engagement with the popular press, turned him into an international reference in psychical research (Nagayama, 2005; Takasuna, 2012; Wu, 2020).

Most of what the Chinese popular press published on *qianliyan* comprised positive reviews of Fukurai and his latest experiments with Japanese mediums. A Japanese article translated into Chinese in 1910 “to provide philosophers and psychologists with materials for research” praised the clairvoyant abilities of Mifune Chizuko (1886–1911)—another of Fukurai's subjects—who during the Russo‐Japanese War (1904–1905) had successfully identified the whereabouts of survivors of a shipwreck (“Qianliyan”, 1911). Others applauded Fukurai's experiments with the two daughters of an amateur hypnotist, both of whom could use their psychic powers to diagnose illness, see through people's bodies, and heal the sick (Zhang, 1913b). In contrast to the attacks Fukurai and his subjects would eventually suffer in the Japanese press (Takasuna, 2012), Chinese newspapers hailed them as examples to be emulated. The fact that the most accomplished Japanese mediums were women also drew the attention of Chinese feminists, who saw this as a resourceful field for modern Chinese women to exploit. Four years after Mifune's death, for example, the influential *Women's Magazine* dedicated five pages to introducing her achievements to Chinese readers. Translated from Japanese by a Chinese female student living in Japan, the article narrates some of the most extraordinary incidents involving her psychic abilities and experiments with Fukurai and his colleague at Kyoto Imperial University, the psychiatrist Imamura Shinkichi. In an endnote, the Chinese translator explains that while science still struggles to clarify the causes of *qianliyan*, this ability has long been described in Buddhist teachings as the sixth sense, the “Bodhisattva's Enlightened Eye” (*Pusa huiyan*) achieved through the practice of “Selflessness” (*wuwo*)—justifying why “psychology has much to learn from Buddhism” (Yinghe, 1915). The alleged similarities between psychical research and Buddhism would be discussed even further by spiritual scientists in the 1920s and 1930s.

Fukurai became an important reference to Chinese spiritual scientists. Several of his articles were rendered into Chinese in the 1910s, with “The Value of Spiritual Science” (*Yanjiu xinling zhi jiazhi*) being particularly well‐known. First published in 1918 and reprinted multiple times thereafter, the lengthy piece outlines the principles of Spiritual Science and the challenges it posed to the dualistic theory of scientific materialism. Thoughtography, Fukurai declared, had challenged psychophysical parallelism, the theory that affirms the correlation of mental and bodily events yet denies any causal interaction between them. Instead, the fact that certain mediums could burn mental images onto photographic plates demonstrated that “matter and mind are intertwined” like the two sides of a coin. “Most natural scientists state that all phenomena work like a machine,” Fukurai lamented, “and if someone dares to use psychic thought (*xinling sixiang*) to clarify them, they simply dismiss their views as pseudoscience, [claiming that] life occurs by chance, death occurs by chance, the world has no purpose and there are no mysteries whatsoever.” The value of Spiritual Science, Fukurai concluded, was not to reject scientific naturalism but rather to supplement it by considering the psychic forces that underpin reality (Fukurai, 1918).

### 2.3 Novels, anecdotes, and comics

Popular media constitute the best source to assess the enduring fascination that hypnotism has exerted on the general public over the past two centuries. Whether its portrayals are accurate or simply perpetuate stereotypes, it is undeniable that novels, films, and visual arts have played a key role in people's understanding of hypnotism, its benefits as well as terrifying powers (Barrett, 2010). In fin‐de‐siècle Japan, Ichiyanagi Hirotaka reveals how popular media, especially fiction, was instrumental in the dissemination of hypnotism—its vocabulary, principles, and application—among the general population (1997). Although no systematic studies have been done so far, a few works have suggested that the same happened in China, particularly through newspapers and magazines (Jia, 2020; Zhang, 2020).

Offering new possibilities for self‐expression, comprehension of the world, and social change, the novel as a genre became an important revolutionary instrument in early‐20th‐century China (Wang, 1997). It is, therefore, no coincidence that a growing number of popular fiction works revolving around psychical abilities popped up in Chinese newspapers over that period, with writers sometimes publicly resorting to these as a means to praise the benefits of hypnotism and urge its immediate dissemination throughout the country (Jia, 2020). Just as other sources, popular media celebrated hypnotism as a simple, cheap, and effective scientific technique able to improve the individual and society as a whole: apart from its application in medicine, it could also enhance learning, help solve mysterious crimes, and even augment filial piety (e.g., Xiaodu, 1914). This wide application of hypnotism beyond individual healthcare was not restricted to East Asia but a global phenomenon at that time (Gauld, 1992; Guillemain, 2006; Pablo, 2003; Priori, 2014).

**Figure 1:**
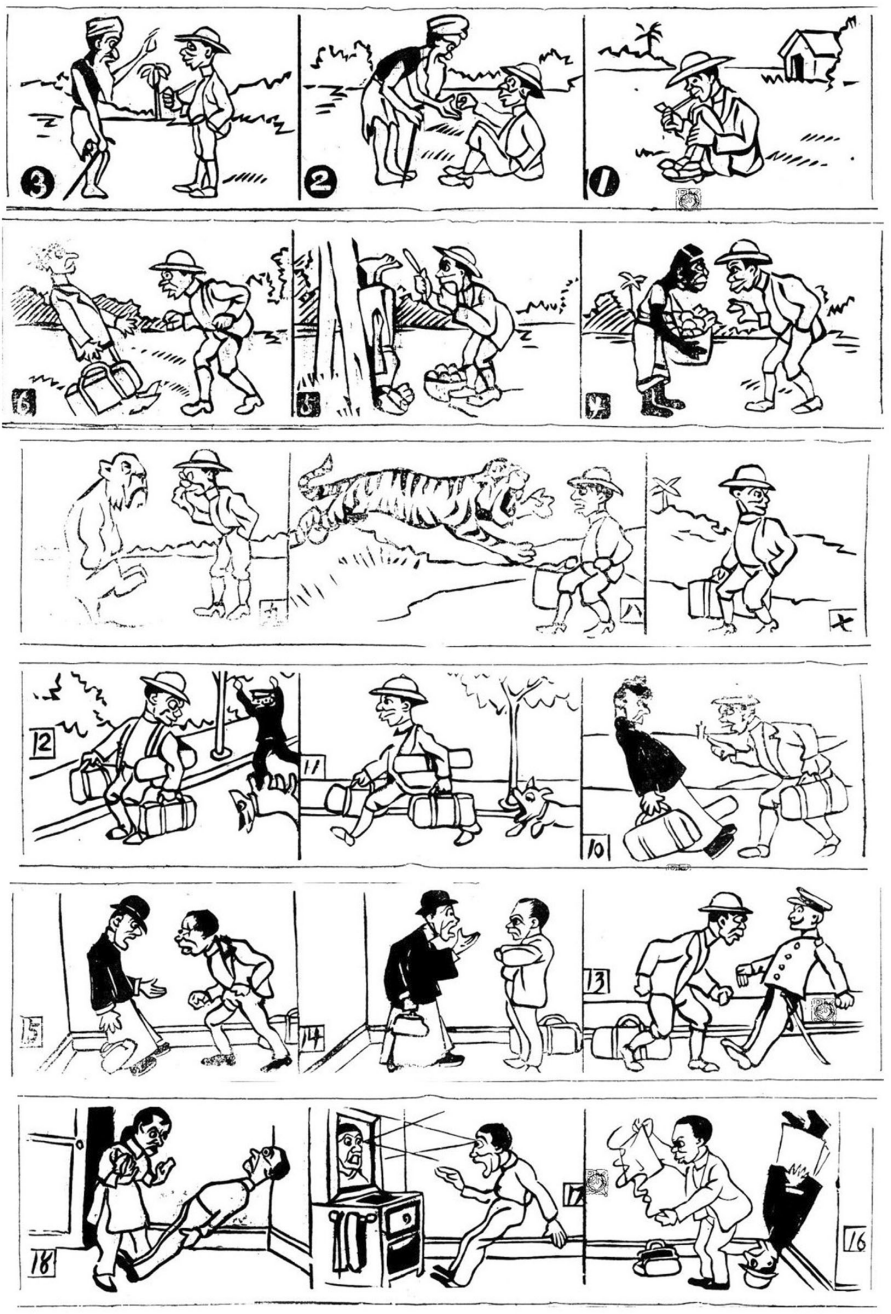
A series of six comic strips depicting the activities of an amateur hypnotist who learned hypnotism from a mysterious Indian beggar. Published in the *Shenghuo ribao* between November 28 and December 3, 1913.

Most of the works analyzed below are Chinese productions inspired by foreign sources. The efficacy of hypnotism and its vast potential was seldom questioned, not even in comic stories—a fact also acknowledged by Barrett (2010) in her research of the Anglo‐American world. A series of six comic strips published in the *Life News*, for example, depicts the trajectory of an amateur hypnotist who used his hypnotic powers to take advantage of others and bypass the police (Figure 1). In the end, the opportunist man returned home so excited and well‐trained that he entered a self‐hypnotic trance by merely looking at himself in the mirror, leaving his wife desperate (Wu, 1913). In another cartoon, a man complains that the manual of hypnotism he bought was so difficult to understand that he fell asleep. During his dreams, however, his “unconscious” discloses that the manual was actually so successful that it had already hypnotized him (Sheyu, 1919). And in another playful dialogue, a daughter asks her mother what hypnotism is, to which the mother says: “if a person is hypnotized, he is unable to control his life.” A few moments passed and the daughter astutely replies: “Doesn't that mean my siblings and I have long fallen into your hypnotic trance, mom?” (Xiaowen, 1915).

Other stories reflected broader trends in Chinese society, like the authority of Japanese hypnotists, the boom of amateur hypnotists, and public performances in Shanghai. An anecdote published in *Vogue Magazine* tells the story of a young Chinese doctor who opened a private clinic in Shanghai after learning hypnotism in Japan. Upon returning to China, he pretended to be Japanese, changing his name and always wearing a Western suit just like a “modern Japanese citizen.” “Today,” he admitted to a client, “it is hard to do business in China if we do not rely on a foreign authority!” (Qian, 1914). Elsewhere, a theatrical play for children narrates the comic story of a young amateur hypnotist who failed to put his friends into the hypnotic trance yet hypnotized himself instead (Banmei, 1915), and from another anecdote published in the same year, we learn the struggles of a Chinese mother who, imitating the stage hypnotists she loved watching in the theater, used the technique to make her baby stop crying at night (Gongyi, 1915).

Children featured prominently in such literary works. Not only were they believed to be among the most suggestible subjects, but they also represented the future of the country and its only hope for survival—a widespread feeling in early‐20th‐century China (Baum, 2017). Moreover, hypnotism promised not only to heal illnesses of the mind and body but also improve moral and civic education just as it had done in Japan (Wu, 2019). In 1917, the illustration of a middle‐aged man hypnotizing a child is accompanied by a written explanation that the teacher did so to find out whether his pupil had stolen money from a classmate. While awaken, the child failed to admit his misconduct, but after being put into a trance, he returned the spoils intact (“Cuimianshu,” 1917). And in the story of another naughty boy, we learn how a Chinese man used hypnotism to give a child a lesson not to disobey his father but become a filial son instead (“Cuimianshu,” 1916). Openly supported by reformers and government officials, this broad application of hypnotism was widely promoted in hundreds of manuals published in Republican China, which offered a wide array of examples and techniques to reshape the Chinese mind from the inside out.

## 3 PSYCHICAL RESEARCH THROUGH BOOKS

### 3.1 Translated books and manuals

Meiji Japan (1868–1912) was held in the highest esteem by early‐20th‐century Chinese elites. This, alongside the similarities between written Chinese and Japanese, led the latter to become by far the most favored foreign language among Chinese intellectuals—and Japan the favorite destination of Chinese students. This fascination with Japan was equally obvious in the realm of publishing. Waves of books on such varied disciplines as law, medicine, engineering, religion, and philosophy were rendered into Chinese from Japanese, comprising the core of everything published on those modern subjects in Republican China (Tian, 2017). In fact, not a few titles written originally in English, French, or German eventually found their way into China through second—if not third‐hand Japanese translations.

Apart from newspapers and magazines, books also became an important vehicle for the dissemination of psychical research in China. The earliest manuals of hypnotism published in Chinese came out by the turn of the century. Most of them consisted of best‐sellers in Japanese translated by Chinese students living (or who had lived) in Japan; many of these manuals enjoyed dozens of reprints in the Republican period, and some remain in print today. It is not difficult to understand why Japan had such a lasting impact on the development of psychical research in China. Psychical research was popularized in Japan years before it drew serious attention from Chinese elites. Consequently, by the time large groups of young Chinese moved to Japan in the 1900s, hundreds of manuals of hypnotism, psychical abilities, and mind‐cure could be easily purchased in bookstores across the country (Ichiyanagi, 1996; Yoshinaga, 2006, 2007).

Preliminary finds reveal that well over 70 manuals of hypnotism and psychical abilities were published in China between 1903 and 1921. This figure would jump to unprecedented heights in the 1920s and 1930s thanks to the emergence of Spiritual Science groups and their active engagement with the printing press.^3^ Just as with stage hypnotism, the earliest individuals involved in Chinese translations of psychical research texts were also foreigners. In 1896, the English missionary and sinologist John Fryer (1839–1928) published a Chinese translation of Henry Wood's seminal *Ideal Suggestion Through Mental Photography* (1893). A prolific New Thought writer, Wood (1834–1909) gained prominence in the United States for his practice of mind‐cure (Braden, 1963). Translated into Chinese as *Zhixin mianbingfa* (Methods for Healing the Heart and Averting Disease), the book combines a range of spiritual and hypnotic suggestions but seems not to have awakened as much interest among Chinese elites as the English version did in the United States.

The thirst for hypnotism and psychic methods began to take hold in China in the first years of the 20th century, being mostly driven by Chinese students in Japan. While studying medicine at Chiba School of Medicine, for instance, the then medical student Wang Nuoyan (1887–1968) sent to print the *Cuimianshu shishufa* (Empirical Methods of Hypnotism), one of the earliest Chinese translations of a Japanese manual of hypnotism. Printed by the Office for Chinese Overseas Students of the Qing Empire in 1903, no copies seem to have survived. Less than 2 years later, a Shanghai‐based Japanese publisher announced the sale of its *Cuimianxue jingli* (Refined Theories of Hypnotism). Advertised as an “occult book” (*qishu*), the manual was translated by Jiang Tun and Wei Dong, two young Chinese who while studying in Japan set up the *Zhongguo cuimian yanjiuhui* (Chinese Institute of Hypnotism) (“Zhongguo conglai,” 1905). Announcements hailed it “the best manual of Japanese hypnotism published so far,” urging everyone “from educators, doctors, politicians, diplomats, judges, and detectives to those interested in the mysteries of the world and the psychology of animals to purchase it” (“Potianhuang,” 1905). The view that hypnotism could benefit both the individual and society is already conspicuous in these early titles, reflecting the global optimism regarding its use at the time.

It was also in 1905 that the Commercial Press published the first edition of the bestselling *Cuimianshu jiangyi* (Lectures on Hypnotism), authored by Huiji shanren, the pseudonym of the prominent Chinese revolutionary Tao Chengzhang (1878–1912). Alongside Cai Yuanpei and others, Tao cofounded the anti‐Qing organization Restoration Society in 1904, which ultimately helped overthrow the Chinese imperial system and establish the Republic of China in 1912. Persecuted by the Qing state, Tao fled to Japan in 1902, where he continued his radical political activities while learning hypnotism under Cai's sponsorship (Jia, 2020). According to his political comrade, Wei Lan (1866–1928), in 1903 Tao invited Chen Daqi—who was studying psychology in Japan—to learn hypnotism together so as to help “eradicate superstition from Chinese society” and “benefit [our] revolutionary activities” (Wei, 1986 [1912], pp. 431–432). Wei's view finds support in the *Lectures*, where Tao shows his enthusiasm regarding the use of hypnotism for memory manipulation, personality change, and developing a “killer instinct,” alongside the cultivation of psychic powers like telepathy and clairvoyance. Others, however, insisted that Tao had engaged in hypnotism only to “make a living through cheating” (Liu, 2014 [1943–1944], p. 170). Whatever his motivation might have been, Tao's life story as a political revolutionary interested in mind‐cure was far from exception among Chinese spiritual scientists^4^—as it would be evident in the membership lists of psychical research associations in the 1920s.

Written in vernacular Chinese, the manual consists of a series of public lectures Tao delivered in Shanghai between 1903 and 1904. Tao explains that while exiled in Japan, he purchased a manual of self‐hypnotism and practiced it with good results. A few months later, he became a pupil of accomplished Japanese hypnotists; “I observed their experiments (*shiyan*) and began to have some realization.” The Chinese audience was excited to hear from Tao that “whereas many countries have been studying [hypnotism], Japan has excelled at it, particularly in its application in education and medicine” (Tao, 1905, p. 1). To Tao, hypnotism should serve as a tool for China's political, social, and scientific development. Divided into 11 chapters, the manual introduces the origins of hypnotism, its principles and methods, furnishing Chinese readers with a Sino‐Japanese glossary that would eventually be integrated into modern Chinese dictionaries. *Lectures* offer detailed instructions for use—like in healing, anesthesia, and parturition—and explain its more remarkable psychic effects. The manual's tremendous success surpassed the author's tragic assassination in 1912. Ten years after it came out for the first time, the manual was already enjoying its 13th edition, and its 24th by 1928, rendering it the most popular guide to hypnotism ever published in China. Advertisements for the manual appeared in the Chinese press all through the late 1930s, praising it as a synthesis of the “latest and most refined books of hypnotism ever compiled,” with all its theories and methods “scientifically proved” (“Xinli qishu,” 1905).

### 3.2 Lectures on mysteries studies

In 1906, the Commercial Press published the first Chinese edition of Inoue Enryō's (1858–1919) *Yōkaigaku‐kōgi* (Lectures on Mystery Studies), a series of lectures Inoue had delivered in Tokyo between 1893 and 1894. Translated by the political activist and education reformer Cai Yuanpei (1868–1940), *Lectures* offers a systematic study on “mysteries” (*yōkai*) intertwined with debates about the role of science and religion in the modernization of Japan. Inoue was one of the most influential Buddhist reformers of Meiji Japan. Ordained as a Buddhist monk at the age of 13, he studied Classical Chinese under the guidance of Confucian scholars, majoring in philosophy at the prestigious Tokyo Imperial University in 1885 (Miura, 2014). His familiarity with Chinese culture would play a pivotal role in the establishment of Mysteries Studies (*yōkaigaku*) in the 1890s and make his works particularly attractive to Chinese readers. *Lectures* had a lasting impact on the development of Spiritual Science, summarizing the central arguments spiritual scientists would uphold later.

Despite the radical material changes seen in fin‐de‐siècle Japan, Inoue believed the Meiji ideal had been accomplished only half‐way. “The other half has not been carried out. Material and technological civilization has already arrived, but nonmaterial and spiritual civilization has not” (cit. Miura, 2014, p. 129). Material reform should thus be accompanied, if not led by a thorough transformation of the Japanese mind. Criticizing the moral decadence of Meiji Japan, Inoue asserted that religion was essential for a modern nation. The fundamental principles of Buddhism, he declared, were consistent with philosophical and scientific truth, and Buddhism could also impede Christian infiltration while enhancing the moral and civic education of his countrymen. But to harness its potential, Buddhism should be reinvented (Schulzer, 2019).

Directly inspired by the SPR, Inoue's study on mysteries was driven by his lifelong mission to eliminate superstition and modernize the Japanese mind. After 10 years of intense investigation, he published *Lectures* as a series of articles (1893–1894) and eventually as a monograph (1897) (Itakura, 1987). According to Cai Yuanpei's diary (1998 [1897]), on May 8, 1901, Cai asked his friend, the editor for the *Eastern Miscellany*, Du Yaquan, to purchase a copy of the *Lectures* for him during his next visit to Japan. On November 10 of the same year, Du invited Cai to translate the text into Chinese, which he completed in 1906. At that time, Cai was already a leading political activist and educational reformer familiar with Inoue's writings (Gildow, 2018). While studying abroad in Japan (1903–1904) and Germany (1907–1911)—where he majored in philosophy, psychology, and art history under the supervision of Karl Lamprecht and Wilhelm Wundt—Cai became directly involved with socialist and anarchist groups. Following the foundation of the Republic of China in 1912, he served as Minister of Education, founding member of the Academia Sinica, and president of Peking University where, alongside Chen Daqi, he oversaw the establishment of China's first psychological laboratory in 1917 (Gao, 2013). In the late 1910s, Cai also showed some interest in the activities of the SoS.

Like other Chinese revolutionaries frustrated over the failure of the Hundred Days of Reform (1898) and admirers of Meiji Japan, Cai believed that Buddhism could save China from political and moral decay, catalyzing the implementation of educational reforms (Gildow, 2018). As a result, Inoue's admonition to “Protect the Nation and Love the Truth”—intertwined with his ideal of a renewed Buddhism free of superstition and focused on education—soon captured the eyes of Cai and his comrades. Inspired by Inoue, in 1900 Cai published *Treatise on Buddhism Protecting the Nation* (*Fojiao huguolun*), and pre‐1903 entries in his diary reveal his enthusiasm for other of Inoue's works on philosophy, psychology, and scientific Buddhism. By the time he became Minister of Education in 1912, 14 works by Inoue had already been translated into Chinese—four of which were dedicated exclusively to Mysteries Studies—including an original introduction to the subject published in 1902 (Wang, 2013).

*Lectures* comprises a lengthy exposition of the field of Mystery Studies. By “mystery,” Inoue referred not simply to the conventional meaning of *yōkai* as “ghost” or “monster” but to all kinds of unusual phenomena not experienced in daily life:

There are many things in the world that people call mysterious or enigmatic. Ordinarily, they end up being made into gods or devils, even though it is difficult to determine whether this is the case or not. [These phenomena] are simply taken as deeds of gods or devils, about whom, today, we do not yet even know if they exist or not. *Moreover, not [even] to ask what mysteries are, cannot in any way be the conduct of a scholar*. Therefore, in the free time of my daily schedule I investigate what [these phenomena] are. I try to ascertain whether they really are gods or devils, or whether there are grounds to consider them differently from the viewpoint of natural science or psychology (Inoue, 1897; tr. Miura, 2014, p. 132; my emphasis)

Inoue described Mysteries Studies as a branch of applied psychology that synthesized the best of all modern academic disciplines. It was devoted to systematizing and investigating the fundamental principle of mysteries. While recognizing the reality of “True Mysteries” (*shinkai*)—those transcendent phenomena beyond scientific scrutiny—Inoue warned that these must not be confused with “False Mysteries” (*karikai*), man‐made phenomena held by the “foolish people” as the actions of the spirit world (Inoue, 1897; tr. Toda, 2018, pp. 128–129). *Lectures* consist of 12 chapters, whose mysteries are systematized into 7 major sections—science, medicine, genuine philosophy, psychology, religious studies, education studies, and miscellany. To write it, Inoue resorted to a copious volume of oral and written accounts. From the late 1880s to the publication of the first article, he traveled around Japan collecting stories about mysterious phenomena, and by 1893 he had also accrued 462 mysterious cases communicated to him via correspondence. His annotated bibliography is even more impressive, including hundreds of titles from Japan, India, and China spanning over 2000 years. As a specialist in Chinese literature, Inoue immediately realized that “many of our country's mysteries come from China, and there are truly very few that we can say are unique to Japan. I estimate that 70% of the mysteries passed down in our country are from China, 20% from India, and 10% unique to Japan” (Inoue, 1897; tr. Toda, 2018, p. 114). Such discovery partly explains the book's later success in China.

Mysteries are, in essence, “confused errors,” whose foremost cause for endurance are “old theories and old stories existing in people's memories and becoming preconceptions.” But even daily news should not be easily believed. Not only do “they arise from a variety of circumstances,” but it is part of human nature to “embellish and elaborate what they see and hear when sharing [those stories] with others” (Inoue, 1897; tr. Toda, 2018, pp. 156–157). Other reasons for mysteries to emerge include the fact they are uncommon matters and humans are curious and full of preconceived notions—which lead them to like or dislike a mysterious story and, consequently, decide to pass it on or not. Just like Cai, Inoue also held that scientific education was the key to a country's modernization. Mysteries are “confused errors,” but not everyone has experienced them. “If there happens to be someone who has actually experienced a mystery, oftentimes they are not a scholar but a fool, not a man but a woman, not a city dweller but a countryside dweller, and not a high‐class but a low‐class person” (Inoue, 1897; tr. Toda, 2018, p. 158). Inoue's Confucian background and advocacy for mass scientific education are evident in this statement.

Nevertheless, Inoue did not identify himself with “extreme skeptics” who rejected the existence of mysteries altogether without investigating their causes:

[If] one holds that mysteries are caused by the functioning of the nerves, then one must explain why the nervous system acts in this way, as well as the nature of nerves and their relationship to the outside world. Furthermore, while the nervous system has the power to produce mysteries, there is no way that they would suddenly arise without a cause (Inoue, 1897; tr. Toda, 2019, p. 64)

To despise a mystery without having properly analyzed it was deemed a behavior as harmful to society as attributing them to supernatural forces. Trying to suppress the propagation of mysteries by force or simply denying their existence, Inoue argued, had already proven as futile as screaming to the four corners of the world that they are fallacies. They are fallacies, but to say so is not enough. Echoing American and European psychical researchers, Inoue's agnostic stance was straightforward: one must not deny the reality of any phenomenon before having thoroughly examined its causes.

Titled *Lectures on Demonology* (*Yaoguaixue jiangyilu*, 1906) in Chinese, Cai's translation was received with clamor by his countrymen.^5^ Reprinted several times over the 20th century, it has been recently hailed as one of the “100 most influential translations in modern China” (Zou, 2008). Inoue's influence on spiritual scientists was profound. The second director of the CIM, Liu Yuchi, graduated from Inoue's Philosophy Academy (*Tetsugakukan*), where among various other subjects, Inoue taught hypnotism and mind‐cure (Nomura, 2013). Likewise, selected translations of *Lectures* frequently appeared in the Institute's magazines. In 1916, for example, the Institute published a short paper by Inoue regarding the universal belief in dragons. Rejecting their existence as celestial beings, Inoue resorted to suggestion, imagination, and scientific ignorance to explain dragons' enduring presence in the minds of the Japanese and Chinese populace (Inoue, 1916). Spiritual scientists shared Inoue's concern about the dangers of superstition, praising his use of science as the best way to eliminate it. Just like him, they would draw heavily on psychology as a means to “naturalize” the causes of phenomena allegedly misattributed by the folk to divine forces (Itakura, 1987), but unlike Inoue, they seemed more optimistic about the existence of abilities like telepathy, clairvoyance, and thoughtography. Their reading of *Lectures* emphasized Inoue's agnostic, naturalistic approach regarding the social and scientific value of investigating psychic effects: no phenomenon should be rejected a priori merely because it does not fit conventional expectations, and it is precisely the lack of examination what makes superstitions flourish (Yu, 1932a). *Lectures'* relevance to psychical research is also seen in the manner it was advertised in the Chinese press: alongside titles of hypnotism, meditation, evolution theory, scientific Buddhism, and Western mediumship (“Cuimianshu,” 1919)—all subjects Inoue and spiritual scientists were fond of (Itakura, 1987).

## 4 PUBLIC LECTURES AND STAGE HYPNOTISM

Public demonstrations played a critical role in the popularization of hypnotism in the late 19th and early 20th centuries. Leading psychical researchers such as William Crookes, Charles Richet, and William James—to name just a few—confessed that their initial interest in the scientific study of psychics arose upon watching the performances of mesmerists, mediums, and hypnotists (Gauld, 1992; Graus, 2014), activities that were equally instrumental in the diffusion of hypnotism and psychical research in Meiji Japan (Ichiyanagi, 1997, 2021; Nagayama, 2005). China was no exception here, with many Chinese intellectuals disclosing that their first contact with psychical research occurred not through printed matter but rather through public performances. Widely advertised in the Chinese press, these performances helped the Chinese public familiarize themselves with the principles of psychical research, seeing with their own eyes—and feeling in their own bodies—the wondrous effects of a hypnotized mind.

The first wave of stage hypnotists arrived in Shanghai from the United States, Western Europe, and Australia by the turn of the century. An article published in the *Shibao* in November 1905 traces the beginnings of stage hypnotism in China back to 1903 when an American hypnotist performed at a foreign theater in Shanghai in front of a crowd of foreign and Chinese spectators (“Guanshiyan,” 1905). Although the hypnotist's name is not specified, it might be referring not to a single person but to the celebrated couple Professor Grossi and Madame Roux, who had previously demonstrated in India, South Africa, Australia, and London (“Tonight,” 1905). Before coming to Shanghai in December 1903, the couple had also made a stir at the Theater Royal in Hong Kong with their performances of “Spiritualism, Magnetism, Hypnotism with Mental Suggestion, Telepathy, Catalepsy and Anasthesy [which] plunged [their] whole audience into mystification” (“Madame Roux and Professor Grossi,” 1903). Professor Grossi and Madame Roux performed in Shanghai for almost 2 weeks. The morning after the couple's first demonstration at the prestigious Lyceum Theater, local newspapers hailed the event as “one of the best entertainments to have ever come to Shanghai” (“Last Night,” 1903). The series of performances, an English‐language newspaper declared, made “wary Shanghailanders” rather shy by seeing a “professor of eccentric exterior [making] his appearance in their midst and [offering] to show them some ‘new thing' in the strange and marvellous.” By “strange and marvellous” it referred, for example, to hypnotically inducing a member of the China Maritime Customs to eat “a candle from the lighted end in the idea that he is digesting chocolate” (“There are hypnotists,” 1903).

Just a few months following the event, in June 1904 another couple, Mr. and Mrs. Robinson Barnett, initiated a series of psychical performances again at the Lyceum Theater, the “second time such a spectacle took place in China.” Although the Hong Kong‐based *South China Morning Post* described their performance as a failure (“Theatre goers,” 1904), the *Shibao* praised the mental abilities of that “young Australian woman” as “far sharper than that of common people” (“Guanshiyan,” 1905). It is also here where we learn about the performances' program through a Chinese reporter who attended the event to “validated its authenticity.” The demonstrations involved Mrs. Barnett and randomly selected members of the audience. In one case, a local Chinese man blindfolded Mrs. Barnett with a piece of red cloth, tied up her arms, hands, and legs to a stake, and sat in front of her holding her knees. The curtains were pulled down, and after being raised again in a matter of seconds, to everyone's astonishment the piece of red cloth was now blindfolding the man, whose Chinese clothes had been exchanged with those of Mrs. Barnett. When the reporter asked for details about the incident, the Chinese man declared that when the curtains closed, “I was suddenly blindfolded with my arms tied up tightly, and I have no idea whatsoever how all that happened.” Speechless, the reporter insisted it would be impossible for all this to occur in such a short time.

In the final demonstration, the reporter tested Mrs. Barnett's telepathic abilities—or, as he explained, how she could “read other people's minds through etheric waves.” “I hid a pen in the corner of the wall and concentrated my mind on another place to test her powers. When she came back to the room and could not find the object, she urged me to concentrate on the place where the object was hidden. I did so and she immediately followed my thoughts, turned towards the corner, and found the pen.” All this, the reporter assured, was due to the “works of the mind” (*xinli zuoyong*). The hypothesis that ether served as a conductor for the transmission of thoughts was particularly welcomed by physical‐psychical scientists like Lodge, Crookes, and Barrett (Noakes, 2019; Raia, 2007). The fact the Chinese reporter knew this theory is not strange as public demonstrations combined entertainment with instruction—let alone that ether was a relatively well‐known concept in China by that time (Wright, 1994). From then to the 1920s, Chinese and foreign elites continued to invite Western stage hypnotists to perform in Shanghai. In 1920, for instance, the director of an American school of hypnotism and his entourage of 60 students set off on a research trip across Singapore, Japan, Shanghai, and Beijing to learn about the “latest Japanese experiments with hypnotism” and recruit eight Chinese to study hypnotism back in New York (“Meiguo,” 1920).

The second wave of stage hypnotists arrived from Japan immediately after Western hypnotists began performing on Chinese soil. Contrary to Westerners, who seemed more interested in offering an “unforgettable spectacle,” the Japanese were concerned with the long‐term benefits that hypnotism and psychical research could offer China, which also explains why the Chinese‐language press tended to describe their activities as “experiments” (*shiyan*) rather than ordinary forms of entertainment. While touching on the activities of Western stage hypnotists, the *Shibao* article actually focused on “the first Japanese to have ever performed in China,” Mr. Nakamizo. Little is known about Nakamizo other than he was a dentist and performed regularly at schools in the Shanghai International Settlement. His demonstrations included somnambulism, distance hypnotism, telepathy, and martial arts, the latter of which illustrated the “relationship between the brain and the physical body” (“Cuimianshu shiyan,” 1905). Nakamizo would also lecture about the history of mesmerism and hypnotism in Europe and the United States, as well as their recent developments in Japan.

Members of the audience took an active part in Nakamizo's demonstrations. In one experiment, he induced a Chinese boy to believe that his hand was a twig, then a knife, also telling him that a matchbox was a pear: the boy then peeled off the “pear” with the “knife” and ate it. In another experiment, Nakamizo hypnotized the editor's friend, piercing several needles through his arm without the man “feeling any apparent pain or spelling a single drop of blood”; this, he explained, “proved the efficacy of hypnotism for anesthesia, a technique widely adopted in Japanese medical universities […] which avoids the dangerous side effects of chemical methods.” Nakamizo also induced a man into hypnotism through the telephone, put a piece of hot coal in another's mouth, and helped a third see objects through solid walls. “For thousands of years,” the Chinese editor lamented, “nobody could explain the rationale behind psychic phenomena. Foolish people believed these to be the action of spirits, pedantic Confucians suspected them as heterodoxy, and even so‐called ‘intellectuals' dismissed all this as fabrication.” Hypnotism, however, confirmed the reality of such manifestations by placing them within the natural realm (“Cuimianshu shiyan,” 1905).

From 1905 to 1920, dozens of Japanese hypnotists continued to perform in Shanghai, either alone or assisted by their Chinese pupils. During the first half of 1910, Nakamura Roshū and Miura Nansei demonstrated at several venues in Shanghai, from medical universities and primary schools to hospitals, hotels, and clubs. Their performances roughly followed the same line as Nakamizo's (“Cuimianshu Xinlixuehui,” 1910). By 1917, Nakamura's wife had already gained much fame thanks to her remarkable abilities of somnambulism, clairvoyance, and telepathy. Reporters who watched their performances welcomed the benefits hypnotism could bring to Chinese politics and military affairs, hailing it as an indispensable technique in the country's modernization endeavor (“Canguan Zhongcun,” 1917). The couple's success as stage hypnotists eventually led them to establish the Eastern Society for Hypnotism (*Dongfang cuimianshu jiangxihui*) in the Shanghai French Concession in the mid‐1910s, which edited manuals and offered a variety of courses to cultivate psychic abilities.

In 1914, another prominent Japanese hypnotist, Suzuki Narao, conducted a series of “experiments” in Shanghai. Chinese newspapers introduced Suzuki as the director of a Japanese psychical research association, with sources in Japanese confirming his identity as a cofounder of the Research Institute of Psychotherapy (*Shinri chiryō kenkyūjo*) (Mimura, 2006). Between May and June, the Ningbo Association in Shanghai and World's Chinese Students' Federation both invited Suzuki to perform. Suzuki's demonstration at the Ningbo Association on May 23, 1914, commenced with an introduction by Hong Chengqi, the Association's director, who explained the reason he invited Suzuki to the stage. “The Chinese are so superstitious,” Hong bemoaned, “that [superstition] is deeply entrenched in their hearts and not easily removed. As a branch of psychology, hypnotism can help eliminate superstition and improve wisdom” (“Cuimianshu yanjiang,” 1914). In the years that followed, the number of Japanese stage hypnotists in China continued to increase, with most of them following the framework set earlier by such figures as Nakamizo, Nakamura, and Suzuki—a mixture of entertainment, first‐hand experience, and didactic lectures.

**Figure 2:**
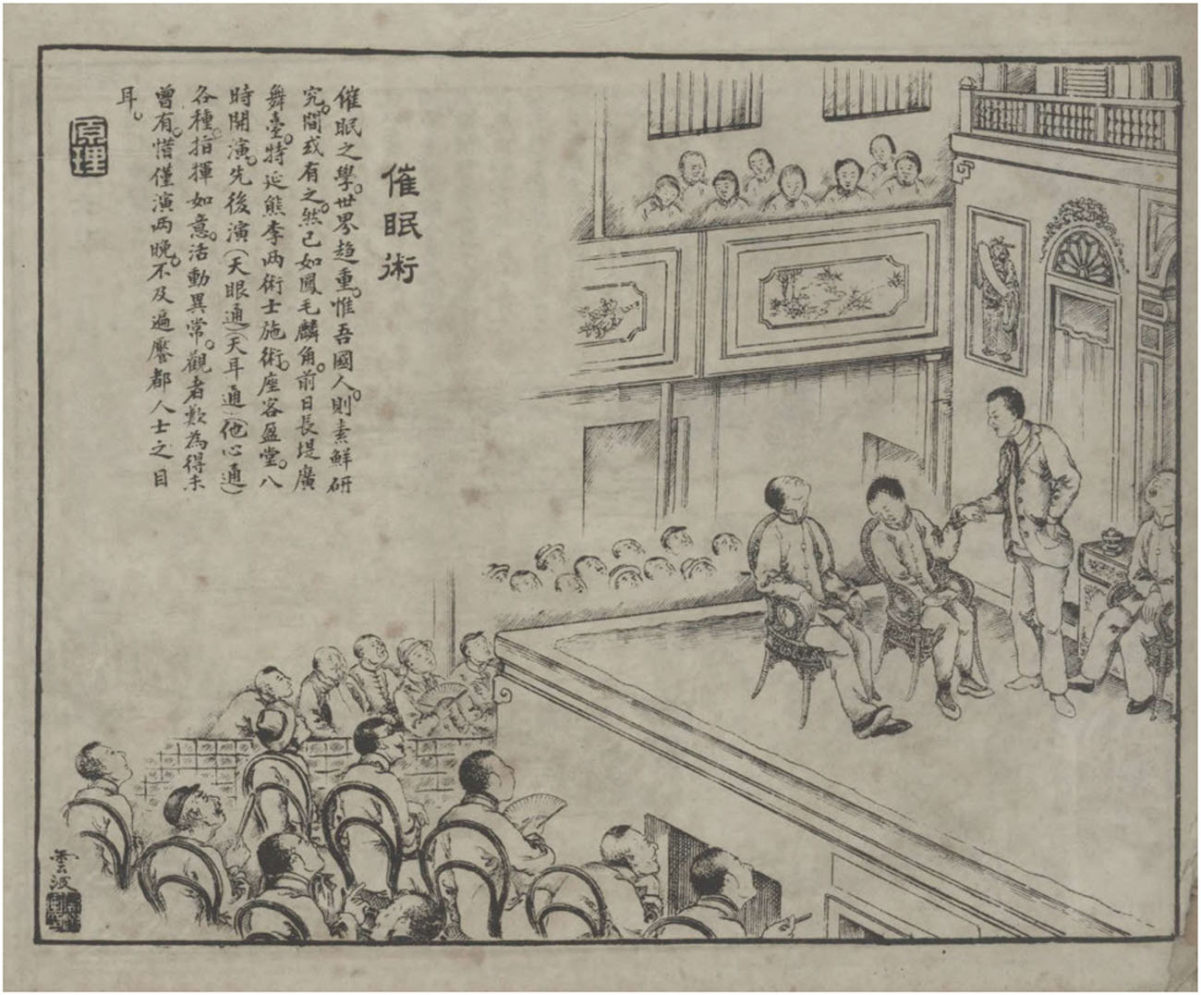
A public performance of hypnotism by a certain Mr. Xiong and Mr. Li in a Shanghai theater. Pay attention to the astonished faces of the audience. Yunbo (1912).

The third and most prosperous wave of stage hypnotists consisted of local Chinese. While they were Chinese in terms of nationality, most of them turned to hypnotism after learning about it in Japan. In 1907, a short text in the *Shibao* announced that a certain “Mr. Ye,” identified as a Chinese student who had just returned from Japan, would demonstrate hypnotism at a Buddhist temple in Kaifeng, Henan province, and was looking for prospective students (“Kaihui jiaoshou,” 1907). Before the 1910s, records of Chinese stage hypnotists appeared only sporadically in the press; in contrast, from the mid‐1910s, the market of stage hypnotism became largely dominated by them. In 1912, the *Current Affairs Pictorial* dedicated a full page to announcing a series of “experiments” by a certain Mr. Xiong and Mr. Li, both of whom arrived in Shanghai from Guangzhou to demonstrate hypnotism, telepathy, and ear‐reading. The words in the short announcement are subsumed by a captivating illustration of a Chinese hypnotist holding the left hand of a recently hypnotized Chinese woman, with two others reclining on a chair in a somnambulic trance (Figure 2). The audience, some with open mouths and others pointing fingers, stare at the performance in awe. The Chinese hypnotist is the only figure in the drawing to be wearing a Western suit—a symbol of modernity in Republican China (Harrison, 2000)—while everyone else is dressed in traditional Chinese clothes. In this missionary‐like scene, advanced Western culture, expressed by the Chinese hypnotist in modern Western attire, is made approachable through the efforts of a young Chinese concerned with the future of his motherland. As the caption laments, “The science of hypnotism is well known worldwide, and the Chinese are the only people still unfamiliar with it!” (Yunbo, 1912).

In 1917, the magazine *Damask* devoted not less than three pages to describing the latest performances of Chen Yanxiang in Shanghai (Bao et al., 1917). A Chinese doctor “who has studied hypnotism for years,” Chen was “a specialist in healing diseases that medicinal drugs and acupuncture cannot treat”—a common saying among traditional talismanic healers as well (Junqueira, 2021, 2022). The demonstrations included healing, entertainment, and psychic effects like hallucination, illusion, and multiple personalities. The article was signed by Chen himself, the martial artist Guo Cuiya, and one of the most influential Chinese hypnotists of the 20th century, Bao Fangzhou (1891–1967).

Given Bao's lasting impact on Spiritual Science, he deserves to be analyzed in more detail in a separate article. Here it suffices to say that during his studies in Japan, Bao also learned hypnotism and established the CHA around 1909. The eventual success of his Association was largely due to the hundreds of performances he gave regularly in Shanghai. In September 1917, for instance, the Chinese Press Club invited all its members to attend a series of demonstrations Bao would deliver at their headquarters in Shanghai (Bao Fangzhou, 1917). A year later, Bao and his cohort staged again at the Grand Shanghai Theatre. Their performance ended with a spectacle by the renowned Chinese magician Han Jingwen (1874–1935), who had previously staged in the United States, Brazil, and Europe (“Shanghai,” 1918). The “self‐experience” dimension of such performances, a local reporter admitted, convinced a vast number of educated Chinese that “the anecdotes about miracles recorded in novels and old texts were not necessarily fabricated but could be achieved by anyone through the latest science of hypnotism” (Yeluo, 1918).

## 5 CONCLUSION

In his global history of phrenology, James Poskett (2019) reveals the paramount role the popular press and public demonstrations assumed in the dissemination of that science throughout the 19th century. As this article has argued, both channels were likewise fundamental in the diffusion of psychical research in China, paving the way for its indigenization as Spiritual Science. The acclaim stage hypnotists received in the 1910s would encourage them to establish some of the earliest and most influential psychical research organizations of the Republican period. By the late 1920s, Shanghai was accommodating over 20 such associations, some of which enjoyed dozens of branches in China and abroad. These institutions allowed spiritual scientists to reach out to an even larger public, positing the transnational identity of Chinese psychical research while ascertaining its usefulness in the salvation of the newly proclaimed Republic—which soon after its establishment in 1912, was reimmersed in civil wars and at risk of collapse. The future of China seemed hopeless, and to many reformers, the creation of a “new‐citizen” (*xinmin*) and “new‐nation” (*xinguo*) required a thorough spiritual revolution (*gexin*) of the Chinese people (Yu, 1932b). While some turned to anarchism, communism, eugenics, Buddhism, or psychoanalysis (Baum, 2017; Huang, 2015; Zarrow, 2005; Zhang, 1992), others hailed psychical research as the ideal tool for social change. Reconciling material and mental realities, it was celebrated as an advanced form of science that no longer relegated the psychical to positions of inferiority and backwardness. To them, the field also allowed a new engagement with science, valuing personal experience as a valid source of knowledge.

Chinese writers emphasized the transnational character of psychical research through the well‐acclaimed reputation of the many European, American, and Japanese scientists and intellectuals engaged in it—actors who, as legitimate sources of authority, would inspire the Chinese to take the field and its controversial objects of study seriously. Being a major concern of Chinese reformers, superstition was categorically denounced as a hindrance to China's path to modernity, leading to decades of countrywide campaigns to eliminate it (Nedostup, 2009). Spiritual Science emerged as a part of and a reaction to these campaigns. To spiritual scientists, phenomena commonly associated by Chinese reformers as “superstition”—such as trance states and exceptional abilities—were neither necessarily fabricated nor caused by divine intervention, but rather produced by an unobstructed mind. Instead of being rejected, these manifestations should be clarified through empirical investigation (Junqueira, in press). Psychical research thus not only naturalized the causes of superstitious phenomena but, as in the case of hypnotism and telepathy, could also harness their beneficial effects for personal and social well‐being.

Similar to Japan, psychical research in China was thoroughly intertwined with stage hypnotism. In their studies of stage magic in fin‐de‐siècle France, Sofie Lachapelle and Graham Jones recount the efforts of French illusionists in “naturalizing” phenomena associated with occult forces. Describing themselves as symbols of modernity and debunkers of superstition, French magicians filled their performances with scientific language, exposing the public to the latest technological breakthroughs and sometimes working closely with psychologists and psychical researchers (Jones, 2018; Lachapelle, 2015). Chinese stage hypnotists also saw themselves as science popularizers; but contrary to those French illusionists, they tended to acknowledge yet condemn the use of hypnotism for entertainment. As such, newspapers presented stage performances primarily as “didactic experiments” aimed to instruct the Chinese public about the potential of hypnotism for the modernization of their country, such as in healthcare, education, and military affairs. Bao Fangzhou himself temporarily worked for the Shanghai police and eventually became a renowned healer of mental illness, with thousands of students from China and abroad.

Wide‐circulation newspapers and popular manuals reached a vast lay audience still unfamiliar with the principles and methods of psychical research. Following the consolidation of Spiritual Science in the early 1920s, the number of newspaper articles and advertisements for hypnotism and psychic abilities would increase exponentially in the Chinese press, promising from self‐healing and the correction of bad habits to the cultivation of exceptional powers and the craft of an “ideal citizen.” Just as it did in Japan (Yoshinaga, 2007), Spiritual Science also prompted a revival of occult arts in Republican China (Junqueira, 2021), traditional methods whose efficacy could be now clarified through the “latest science of psychical research.” Contrary to studies focused on the Euro‐American context, still little is known about the broader impact of psychical research in the making of modern China. It is clear, however, that it would become a catalyst for the dissemination of psychotherapy and mind‐cure across the country, with spiritual scientists being particularly interested in criminal and crowd psychology.

Reform‐minded elites pursued psychical research as an arena for working out historical, social, and spiritual dislocations, hailing it as the “science that will save China” (Yu, 1932b). Spiritual Science flourished as the Chinese expression of a wave of transnational movements dissatisfied with reductive materialism yet concerned with the future of science, humanity, and its place in the cosmos. By the early 1930s, the movement had already become a central channel through which new understandings of the body and mind spread across China from abroad, merging with native knowledge in unusual and creative ways. What was the cultural significance of psychical research in Republican China? To what extent did it open up new possibilities for the Chinese to make sense of old beliefs in a modern world? And how does the history of Spiritual Science contribute to the global histories of science‐making and science communication in general (Poskett, 2019)—and mind–body medicine in particular (Harrington, 2008)? Moving between macro and micro scales, this paper has focused on the introductory phase of psychical research in China. Future studies will be able to shed further light on the consolidation of Spiritual Science within the country's struggle for modernity.

## Data Availability

Data sharing is not applicable to this article as no new data were created or analyzed in this study.

## Endnotes

1 The CIM and CHA were founded in Yokohama and Kobe, respectively, in the mid‐1910s, moving their headquarters to Shanghai only in the early 1920s.

2 Additions within brackets are passages from the Chinese version not seen in the English article.

3 For an in‐progress list of publications, please visit https://luisfbj.com/publications/

4 As Zhang (1992) has noted, there was a rising interest in the psy‐disciplines among Republican intellectuals who saw these as catalysts for their revolutionary activities.

5 Although Cai translated six of the eight volumes of *Lectures*, five of them were destroyed by a fire at Du Yaquan's printing shop. Ultimately, only the first volume, “Outline” (*zonglun*), was printed.

## References

[jhbs22236-bib-0001] Abbott, L. J. (1918). Shengsijie zhi goutong [communications between the worlds of the living and the dead]. *Dongfang zazhi*, *15*(1).

[jhbs22236-bib-0002] Banmei . (1915). Cuimianshu [hypnotism]. Shaonian, 5(7), 1–14.

[jhbs22236-bib-0003] Bao, F. Z. , Chen, T. X. , & Guo, C. Y. (1917). Zhi Chen Tingxiang jun yanshi cuimianshu [Mr. Chen Tingxiang performs hypnotism]. Zhijin, 52–54.

[jhbs22236-bib-0004] Bao Fangzhou shiyan cuimianshu [Bao Fangzhou demonstrates hypnotism]. (1917, 24 September). *Minguoribao.*

[jhbs22236-bib-0005] Barrett, D. (2010). Hypnosis in popular media. In D. Barrett (Ed.), Hypnosis and hypnotherapy (pp. 77–96). Praeger.

[jhbs22236-bib-0006] Barrett, W. F. (1918). The deeper issues of psychical research. Contemporary Review, 113, 169–179.

[jhbs22236-bib-0007] Barrett, W. F. , Gurney, E. , & Myers, F. W. H. (1882). First report of the literary committee. Proceedings of the Society for Psychical Research, 1, 116–155.

[jhbs22236-bib-0008] Baum, E. (2017). Healthy minds, compliant citizens: The politics of “mental hygiene” in Republican China, 1928–1937. Twentieth‐Century China, 42(3), 215–233.

[jhbs22236-bib-0010] Braden, C. S. (1963). Spirits in rebellion: The rise and development of new thought. Southern Methodist University Press.

[jhbs22236-bib-0011] Cai, Y. P. (1998). 1897. *Cai Yuanpei riji* [Cai Yuanpei's diary] (Vol. 15). Zhejiang jiaoyu chubanshe.

[jhbs22236-bib-0012] Canguan Zhongcun Luzhou shiyan cuimianshu [attending Nakamura Roshū's experiment of hypnotism]. (1917, June 4). *Shibao.*

[jhbs22236-bib-0013] Chang, K. M. (2017). Ceaseless generation: Republican China's rediscovery and expansion of domestic vitalism. Asia Major, 30(2), 101–131.

[jhbs22236-bib-0014] Chen, D. Q. (1919). Xinling xianxiang lun [treatise on psychic phenomena]. Beijing daxue rikan.

[jhbs22236-bib-0015] Cuimianshu [hypnotism]. (1916). Zhonghua tongzijie , 22, 42–48.

[jhbs22236-bib-0131] Cuimianshu. (1917, June 4). *Minguo ribao*.

[jhbs22236-bib-0016] Cuimianshu . (1919). Dongfang zazhi , 16(1).

[jhbs22236-bib-0017] Cuimianshu shiyan [demonstration of hypnotism]. (1905, November 26). *Shibao.*

[jhbs22236-bib-0018] Cuimianshu Xinlixuehui shiyan zhilue [records of a hypnotism experiment at the Society for Psychology]. (1910, February 28). *Xinwenbao*.

[jhbs22236-bib-0019] Cuimianshu yanjiang dahui [Lectures on hypnotism]. (1914, June 9). *Shenghuo ribao*.

[jhbs22236-bib-0020] Ding, F. B. (1915). Qianliyan. Zhongxiyi xuebao, 6(4), 1–7.

[jhbs22236-bib-0021] Driesch, H. (1923). Jindai xinlixuezhong zhi feizijue ji xiazixue wenti [the problem of the unconscious and subconscious in modern psychology]. Dongfang zazhi, 20, 8.

[jhbs22236-bib-0022] Ferguson, C. (2020). The case of Fletcher: Shell shock, spiritualism, and Oliver Lodge's Raymond. In J. Mussell , & G. Gooday (Eds.), A pioneer of connection: Recovering the life and career of oliver lodge. University of Pittsburgh Press.

[jhbs22236-bib-0023] Fukurai, T. (1918). Yanjiu xinling zhi jiazhi [the value of spiritual science]. Jiaoyu gongbao, 5(9), 122–133.

[jhbs22236-bib-0024] Gao, Z. (2013). The emergence of modern psychology in China, 1876‐1929. Annual Review of Critical Psychology, 10, 293–307.

[jhbs22236-bib-0128] Gauld, A. (1992). A history of hypnotism. Cambridge University Press.

[jhbs22236-bib-0025] Gildow, D. (2018). Cai Yuanpei (1868–1940), religion, and his plan to save China through Buddhism. Asia Major, 31(2), 107–148.

[jhbs22236-bib-0026] Gongyi . (1915). Yumu zhi cuimianshu [my mom and hypnotism]. Libailiu, 78, 34–39.

[jhbs22236-bib-0027] Graus, A. (2014). Hypnosis in Spain (1888–1905): From spectacle to medical treatment of mediumship. Studies in History and Philosophy of Science Part C, 48, 85–93. 10.1016/j.shpsc.2014.07.002 25127318

[jhbs22236-bib-0028] Guanshiyan cuimianshu xuji [experiments of hypnotism: continuation]. (1905, November 28). *Shibao*.

[jhbs22236-bib-0029] Guillemain, H. (2006). La méthode Coué. Historie d'une pratique de guérison au XXe siècle. Seuil.

[jhbs22236-bib-0127] Hammerstrom, E. J. (2015). The science of Chinese Buddhism: Early twentieth‐Century Engagements. Columbia University Press.

[jhbs22236-bib-0030] Harrington, A. (2008). The cure within: A history of mind‐body medicine. W.W. Norton.

[jhbs22236-bib-0133] Harrison, H. (2000). The making of the Republican citizen: Political ceremonies and symbols in China, 1911–1929. Oxford University Press.

[jhbs22236-bib-0031] Hazelgrove, J. (1999). Spiritualism after the Great War. Twentieth Century British History, 10(4), 404–430.

[jhbs22236-bib-0032] Hickey, W. S. (2019). Mind cure: How meditation became medicine. Oxford University Press.

[jhbs22236-bib-0033] Hu, Y. Z. (1917a). Guanwangshu yi [spirit‐communications I]. *Dongfang zazhi*, 14(5).

[jhbs22236-bib-0034] Hu, Y. Z. (1917b). Guanwangshu er [spirit‐communications II]. Dongfang zazhi, 14(6).

[jhbs22236-bib-0035] Hu, Y. Z. (1921). Mixin yu jindai sixiang [superstition and modern thought]. *Dongfang zazhi* , 18(5).

[jhbs22236-bib-0036] Huang, K. W. (2015). Mixin guannian de qiyuan yu yanbian: Wusi kexueguan de zaifanxing [the origins and evolution of the concept of superstition]. Dongya guannianshi jikan, 9, 153–226.

[jhbs22236-bib-0037] Ichiyanagi, H. (1996). Meijiki kankō no shinreigaku kanren shoseki to sono shūhen [a list of psychical research books published in Meiji Japan]. Nagoya kindai bungaku kenkyū, 17, 29–37.

[jhbs22236-bib-0038] Ichiyanagi, H. (1997). Saiminjutsu no Nihon kindai [hypnotism in modern Japan]. Seikyūsha.

[jhbs22236-bib-0039] Ichiyanagi, H. (2021). Kokkurisan to senrigan Nihon kindai to shinreigaku [Kokkurisan and senrigan: Psychical research in modern Japan]. Seikyūsha.

[jhbs22236-bib-0040] Inoue, E. (1897). Yaoguaixue jiangyilu [lectures on demonology] (Cai, Y.P., Trans., 1906). Shangwu yinshuguan.

[jhbs22236-bib-0041] Inoue, E. (1916). Xuelishang zhi long [dragons from the perspective of science]. Xinling , 1(4).

[jhbs22236-bib-0042] Itakura, K. (1987). Kawaridane no kagakushatachi [eccentric scientists]. Kasetusha.

[jhbs22236-bib-0009] Junqueira, L. F. B. (2021). Revealing secrets: Talismans, healthcare and the market of the occult in early twentieth‐century China. Social History of Medicine, 34(2), 1068–1093. 34899068 10.1093/shm/hkab035 PMC8653939

[jhbs22236-bib-0137] Junqueira, L. F. B. (2022). Numinous herbs: Stars, spirits and medicinal plants in Late Imperial China. In V. Lo , & M. Stanley‐Baker (Eds.), Routledge handbook of Chinese medicine (pp. 456–472). Routledge.

[jhbs22236-bib-0043] Junqueira, L. F. B. (in press). Psychical research and the redefinition of superstition in Republican China. In E. Baum , & A. Wu (Eds.), Uncanny beliefs: Superstition in modern China.

[jhbs22236-bib-0044] Jia, L. Y. (2020). Cuimianshu zai jindai Zhongguo de chuanbo (1839‐1911) [the diffusion of hypnotism in modern China]. Kexue wenhua pinglun, 17(3), 54–70.

[jhbs22236-bib-0136] Jones, G. M. (2018). Magic's reason: An anthropology of analogy. University of Chicago Press .

[jhbs22236-bib-0045] Kaihui jiaoshou cuimianshu zhi keyi [Opening a society for teaching hypnotism]. (1907, December 5). *Shibao*.

[jhbs22236-bib-0046] Lachapelle, S. (2005). Attempting science: The creation and early development of the Institut Métapsychique International in Paris, 1919–1931. Journal of the History of the Behavioral Sciences, 41(1), 1–24. 15635703 10.1002/jhbs.20061

[jhbs22236-bib-0047] Lachapelle, S. (2015). Conjuring science: A history of scientific entertainment and stage magic in modern France. Palgrave Macmillan.

[jhbs22236-bib-0048] Lagerwey, J. (2019). Paradigm shifts in early and modern Chinese religion: A history. Brill.

[jhbs22236-bib-0049] Laing, S. (1885). Modern science and modern thought. Chapman and Hall.

[jhbs22236-bib-0050] Lamont, P. (2012). The making of extraordinary psychological phenomena. Journal of the History of the Behavioral Sciences, 48(1), 1–15. 25363382 10.1002/jhbs.21516

[jhbs22236-bib-0051] Last Night, at the Lyceum. (1903, December 16). *The North‐China Daily News*.

[jhbs22236-bib-0052] Li, S. C. (1920). Kexue yu zhexue zongjiao sanzhe zhi leisidian [science, philosophy and religion: Three common points]. *Xinfojiao*, *2*(3).

[jhbs22236-bib-0053] Li, S. C. (1921). Zongjiaolun [on religion]. *Dongfang zazhi*, *18*(10).

[jhbs22236-bib-0054] Li, S. C. (1923). Foxue yu rensheng [Buddhism and life]. *Dongfang zazhi*, *20*(24).

[jhbs22236-bib-0055] Liu, Y. Z. (2014). Liu Yazi zishu [Memoirs of Liu Yazi]. Qunyan chubanshe.

[jhbs22236-bib-0134] Lodge, O. (1916). Raymond, or life and death: With examples of the evidence for survial of memory and affection after death. Methuen & Co.

[jhbs22236-bib-0056] Luckhurst, R. (2002). The invention of telepathy, 1870‐1901. Oxford University Press.

[jhbs22236-bib-0057] Luo, L. (1918). Xinling yanjiu zhi jinjing [advances in psychical research]. *Dongfang zazhi*, *15*(9).

[jhbs22236-bib-0058] Madame Roux and Professor Grossi. (1903, November 26). *South China Morning Post*.

[jhbs22236-bib-0059] Meiguo cuimianshujia jianglai Hua [American hypnotist is coming to China]. (1920, March 2). *Minguo ribao*.

[jhbs22236-bib-0060] Mimura, S. (2006). Mimura Seizaburō nikki 15. Engeki kenkyū, 30, 45–165.

[jhbs22236-bib-0061] Miura, S. (2014). Inoue Enryo's Mystery Studies. International Inoue Enryo Research, 2, 119–154.

[jhbs22236-bib-0062] Mülberger, A. , (Ed.). (2016). Los límites de la ciencia. Espiritismo, hipnotismo y el estudio de los fenómenos paranormales (1850–1930). Consejo Superior de Investigaciones Científica.

[jhbs22236-bib-0063] Myers, F. W. H. (1903). Human personality and its survival of bodily death. Longmans.

[jhbs22236-bib-0064] Nagayama, Y. (2005). Senrigan jiken Kagaku to okaruto no Meiji Nihon [The senrigan affair: Science and the occult in Meiji Japan]. Heibonsha shinsho.

[jhbs22236-bib-0065] Nedostup, R. (2009). Superstitious regimes: Religion and the politics of Chinese modernity. Harvard University Asia Center.

[jhbs22236-bib-0066] Noakes, R. (2019). Physics and psychics: The occult and the sciences in modern Britain. Cambridge University Press.

[jhbs22236-bib-0067] Nomura, H. (2013). Inoue Enryō ni okeru saiminjutsu to meisōhō [Inoue Enryō's view of hypnotism and meditation]. Eco Philosophy Research, 7, 21–30.

[jhbs22236-bib-0068] Pablo, Á. G. (2003). El hipnotismo en la España del primer tercio del siglo XX. In L. Montiel , & Á. G. Pablo (Eds.), En ningún lugar en parte alguna: Estudios sobre la historia del magnetismo animal y del hipnotismo (pp. 229–297). Frenia.

[jhbs22236-bib-0069] Palmer, D. A. (2007). Qigong fever: Body, science, and utopia in China. Columbia University Press.

[jhbs22236-bib-0070] Poskett, J. (2019). Materials of the mind: Phrenology, race, and the global history of science, 1815‐1920. University of Chicago Press.

[jhbs22236-bib-0071] Potian huangzhi zhenji [a precious book without precedent!]. (1905, August 27). *Shibao*.

[jhbs22236-bib-0072] Priori, M. (2014). Do outro lado: a história do sobrenatural e do espiritismo. Planeta.

[jhbs22236-bib-0073] Ptak, R. (2017). Qianliyan und Shunfeng'er in xiaoshuo und anderen Texten der Yuan‐ und Ming‐Zeit. In B. Hoster (Ed.), Rooted in hope: China—Religion—Christianity. Monumenta Serica Monograph Series (Vol. 68, pp. 571–596). Routledge.

[jhbs22236-bib-0074] Qian, X. R. (1914). Cuimianshu zhibing [hypnotherapy]. Fanhua zazhi, 4, 191–194.

[jhbs22236-bib-0075] Qianliyanzhi qiyan [Strange experiments with *qianliyan*]. (1910, December 23). *Shibao*.

[jhbs22236-bib-0135] Qianliyan zhi yanjiuhui [scientific societies for the study of *qianliyan*]. (1911). *Tongwenbao*, *(464)*, 11.

[jhbs22236-bib-0076] Raia, C. G. (2007). From ether theory to ether theology: Oliver Lodge and the physics of immortality. Journal of the History of the Behavioral Sciences, 43(1), 18–43. 17205538 10.1002/jhbs.20207

[jhbs22236-bib-0077] Schulzer, R. (2019). Inoue Enryō: A philosophical portrait. SUNY Press.

[jhbs22236-bib-0078] Sera‐Shriar, E. (2022). Psychic investigators: Anthropology, modern spiritualism, and credible witnessing in the late Victorian age. University of Pittsburgh Press.

[jhbs22236-bib-0079] Shamdasani, S. (2015). ‘S.W.’ and C.G. Jung: Mediumship, psychiatry and serial exemplarity. History of Psychiatry, 26(3), 288–302. 26254128 10.1177/0957154X14562745

[jhbs22236-bib-0080] Shanghaixiyuan shiyan cuimianshu [demonstration of hypnotism at a Western theater in Shanghai]. (1918, January 4). *Shibao*.

[jhbs22236-bib-0081] Shen, G. Y. (2016). Scientism in the twentieth century. In V. Goossaert , J. Kiely , & J. Laferwey (Eds.), Modern Chinese religion II, 1850–2015 (pp. 89–137). Brill.

[jhbs22236-bib-0082] Sheyu . (1919). Cuimianshu [hypnotism]. Huaji huabao, 1(1), 19.

[jhbs22236-bib-0083] Shijie shenmi zhi yanjiu. (1916). *Dongfang zazhi, 13*(8).

[jhbs22236-bib-0084] Sommer, A. (2012). Psychical research and the origins of American psychology: Hugo Münsterberg, William James and Eusapia Palladino. History of the Human Sciences, 25(2), 23–44. 23355763 10.1177/0952695112439376 PMC3552602

[jhbs22236-bib-0085] Sommer, A. (2013a). *Crossing the boundaries of mind and body: Psychical research and the origins of modern psychology* [PhD dissertation, University College London].

[jhbs22236-bib-0086] Sommer, A. (2013b). Normalizing the supernormal: The formation of the “Gesellschaft fur Psychologische Forschung” (“Society for Psychological Research”), c. 1886‐1890. Journal of the History of the Behavioral Sciences, 49(1), 18–44. 23169462 10.1002/jhbs.21577 PMC3580885

[jhbs22236-bib-0087] Takasuna, M. (2012). The Fukurai affair: Parapsychology and the history of psychology in Japan. History of the Human Sciences, 25(2), 149–164.

[jhbs22236-bib-0088] Tao, C. Z. (1905). Cuimianshu jiangyi [Lectures on hypnotism]. Shangwu yinshuguan.

[jhbs22236-bib-0089] Tao, H. Y. (2013). *Dongfang zazhi yanjiu (1904–1949) xiandai wenhua de shengchangdian [A study of the Eastern Miscellany: The genesis of modern culture]*. [PhD dissertation, Nanjing University].

[jhbs22236-bib-0090] Theatre goers disgusted. (1904, June 24). *South China Morning Post*.

[jhbs22236-bib-0091] There are hypnotists and hypnotists. (1903, December 18). *The North‐China Daily News.*

[jhbs22236-bib-0092] Tian, Y. (2017). Minguo shiqi hanyi riwen tushu de chuban [the translation of Japanese books into Chinese during the Republican period]. Zhongguo chubanshi yanjiu, 2, 114–129.

[jhbs22236-bib-0093] Toda, D. L. (2018). Outline of Mysteries Studies: Part I. International Inoue Enryo Research, 6, 93–175.

[jhbs22236-bib-0094] Toda, D. L. (2019). Outline of Mysteries Studies: Part II. International Inoue Enryo Research, 7, 32–95.

[jhbs22236-bib-0095] Tonight. (1905, October 31). *The Sydney Morning Herald.*

[jhbs22236-bib-0096] Treitel, C. (2004). A science for the soul: Occultism and the genesis of the German Modern. Johns Hopkins University Press.

[jhbs22236-bib-0097] Wang, D. D. W. (1997). Fin‐de‐siècle splendor: Repressed modernities of late Qing fiction, 1848‐1911. Stanford University Press.

[jhbs22236-bib-0098] Wang, Q. (2013). A comparative study of religious thought in the work of Cai Yuanpei and Inoue Enryo. International Inoue Enryo Research, 1, 37–48.

[jhbs22236-bib-0099] Wang, Z. S. (1914). Qiannianyan zhi kexue jieshi [a scientific explanation for *qian'nianyan*]. Dongfang zazhi, 11(4).

[jhbs22236-bib-0100] Wei, L. (1986). Tao Huanqing xiansheng xingshu [a biography of Tao Huanqing]. In Z. J. Yang (Ed.), Tao Chengzhang ji. Zhonghua shuju.

[jhbs22236-bib-0101] Weilaishenghuo zhi xinjieshi [a new explanation for the afterlife]. (1920). *Dongfang zazhi, 17*(6).

[jhbs22236-bib-0132] Wood, H. (1893). Ideal suggestion through mental photography: A restorative system for home and private use, preceded by a study of the laws of mental healing. Lee & Shepard.

[jhbs22236-bib-0102] Wright, D. (1994). Tan Sitong and the ether reconsidered. Bulletin of the School of Oriental and African Studies, 57(3), 551–575.

[jhbs22236-bib-0130] Wu, X. (1913). Cuimianshu. *Shenghuo ribao*, November 28, 29, 30 to December 1, 2, 3.

[jhbs22236-bib-0103] Wu, Y. (2017). Techniques for nothingness: Debate over the comparability of hypnosis and Zen in early‐twentieth‐century Japan. History of Science, 56(4), 470–496. 29219000 10.1177/0073275317743120

[jhbs22236-bib-0104] Wu, Y. (2019). The moral power of suggestion: A history of suggestion in Japan, 1900–1930. Journal of the History of the Behavioral Sciences, 55(1), 21–39. 30508292 10.1002/jhbs.21944

[jhbs22236-bib-0105] Wu, Y. (2020). Seeking double personality: Nakamura Kokyō's work in abnormal psychology in early 20th‐century Japan. Journal of the History of the Behavioral Sciences, 56(4), 258–277. 32594523 10.1002/jhbs.22045

[jhbs22236-bib-0106] Xiaodu . (1914). Cuimianshu [hypnotism]. Wutongyuan, 14, 14–17.

[jhbs22236-bib-0107] Xiaowen . (1915). Qize cuimianshu [hypnotism: seven principles]. Shanghai, 1(1), 232.

[jhbs22236-bib-0109] Xinliqishu [Psychic arts]. (1905, October 19). *Shibao.*

[jhbs22236-bib-0110] Yeluo. (1918, January 8). Zhongguo jingshen yanjiuhui shiyan cuimianshu erzhi [the Chinese Hypnotism Association demonstrates hypnotism: Part II]. *Shibao*.

[jhbs22236-bib-0111] Yinghe . (1915). Qianliyan. *Funü zazhi* , 1(2), 6–10.

[jhbs22236-bib-0112] Yoshinaga, S. I. , (Ed.). (2006). Saiminjutsu no reimei Kindai Nihon rinshōshinri no tanjō *[the dawn of hypnotism and the birth of clinical psychology in modern Japan]* . Kuresushuppan.

[jhbs22236-bib-0113] Yoshinaga, S. I. (2007). *Seishin no chikara: Minkan seishinryōhō no shisō* [the power of the mind: A history of Japanese mind‐cure]. Jintai kagaku, 16(1), 9–21.

[jhbs22236-bib-0115] Yu, P. K. (1922). Xinling yanjiu [on spiritual science]. Xinling, 25, 5–8.

[jhbs22236-bib-0116] Yu, P. K. (1932a). Yanjiu xinling shi yizhong mixin de gongzuo me [is spiritual science a kind of superstition?]. Xinling wenhua, zhuanhao, 9–10.

[jhbs22236-bib-0117] Yu, P. K. (1932b). Zhongguo xinling yanjiuhui chuangli ershizhounian jinian [the 20th anniversary of the Chinese Institute of Mentalism]. *Xinling wenhua, qiuhao*, n.p.

[jhbs22236-bib-0118] Yunbo . (1912). Cuimianshu [hypnotism]. Shishi huabao , p. 14.

[jhbs22236-bib-0119] Zarrow, P. (2005). China in war and revolution, 1895‐1949. Routledge.

[jhbs22236-bib-0120] Zhang, B. Y. (2020). Jingshen de fudiao Jindai Zhongguo de cuimianshu yu dazhongkexue [hypnotism and popular science in modern China]. Lianjing chuban gongsi.

[jhbs22236-bib-0121] Zhang, J. Y. (1992). Psychoanalysis in China: Literary transformations, 1919‐1949. Cornel University Press.

[jhbs22236-bib-0122] Zhang, X. C. (1913a). Qianliyan zhi kexue jieshi [a scientific explanation for *qianliyan*]. Dongfang zazhi , 10(7).

[jhbs22236-bib-0129] Zhang, X. C. (1913b). Nihon xin qianliyan chuxian [new cases of *qianliyan* in Japan]. Dongfang zazhi, 10(4).

[jhbs22236-bib-0123] Zheng, G. (2018). Biandong shehui xia de xinyang fenhua Shanghai lingxuehui yanjiu [A study of the Shanghai Society of Spiritualism]. Zhongghuo shehui kexue chubanshe.

[jhbs22236-bib-0124] Zhongguo conglai weiyou zhi qishu [an occult art unknown to China]. (1905, May 23). *Shibao*.

[jhbs22236-bib-0125] Zou, Z. H. (2008). Yingxiang Zhongguo jindai shehui de yibaizhong yizuo *[the top‐100 most influential translated books in modern China]* . Zhongguo duiwai fanyi chubanshe.

